# From bacterial killing to immune modulation: Recent insights into the functions of lysozyme

**DOI:** 10.1371/journal.ppat.1006512

**Published:** 2017-09-21

**Authors:** Stephanie A. Ragland, Alison K. Criss

**Affiliations:** Department of Microbiology, Immunology, and Cancer Biology, University of Virginia, Charlottesville, Virginia, United States of America; Stony Brook University, UNITED STATES

## Abstract

Lysozyme is a cornerstone of innate immunity. The canonical mechanism for bacterial killing by lysozyme occurs through the hydrolysis of cell wall peptidoglycan (PG). Conventional type (c-type) lysozymes are also highly cationic and can kill certain bacteria independently of PG hydrolytic activity. Reflecting the ongoing arms race between host and invading microorganisms, both gram-positive and gram-negative bacteria have evolved mechanisms to thwart killing by lysozyme. In addition to its direct antimicrobial role, more recent evidence has shown that lysozyme modulates the host immune response to infection. The degradation and lysis of bacteria by lysozyme enhance the release of bacterial products, including PG, that activate pattern recognition receptors in host cells. Yet paradoxically, lysozyme is important for the resolution of inflammation at mucosal sites. This review will highlight recent advances in our understanding of the diverse mechanisms that bacteria use to protect themselves against lysozyme, the intriguing immunomodulatory function of lysozyme, and the relationship between these features in the context of infection.

## Introduction

Ubiquitously encoded in the genomes of the animal kingdom, lysozyme is a conserved antimicrobial protein that is critical to host defense. All lysozymes share the ability to hydrolyze bacterial cell wall peptidoglycan (PG) and have a similar overall structure [1]. The following 3 types of lysozymes have been described based on their amino acid sequence and biochemical properties: chicken or conventional type (c-type), goose type (g-type), and invertebrate type (i-type). Comparisons of lysozymes across the animal kingdom have been extensively reviewed elsewhere [1]. In mammals, lysozyme is found in abundance in the blood and liver, in secretions, including tears, urine, saliva, and milk, at mucosal surfaces (where it can reach concentrations as high as 1 mg/ml), and in professional phagocytes, including macrophages, neutrophils, and dendritic cells [1, 2]. Lysozyme is present in phagocyte-like cells in nonmammalian organisms as well, suggesting that lysozyme plays a conserved role in host defense across the animal kingdom [1, 3].

**Fig 1:**
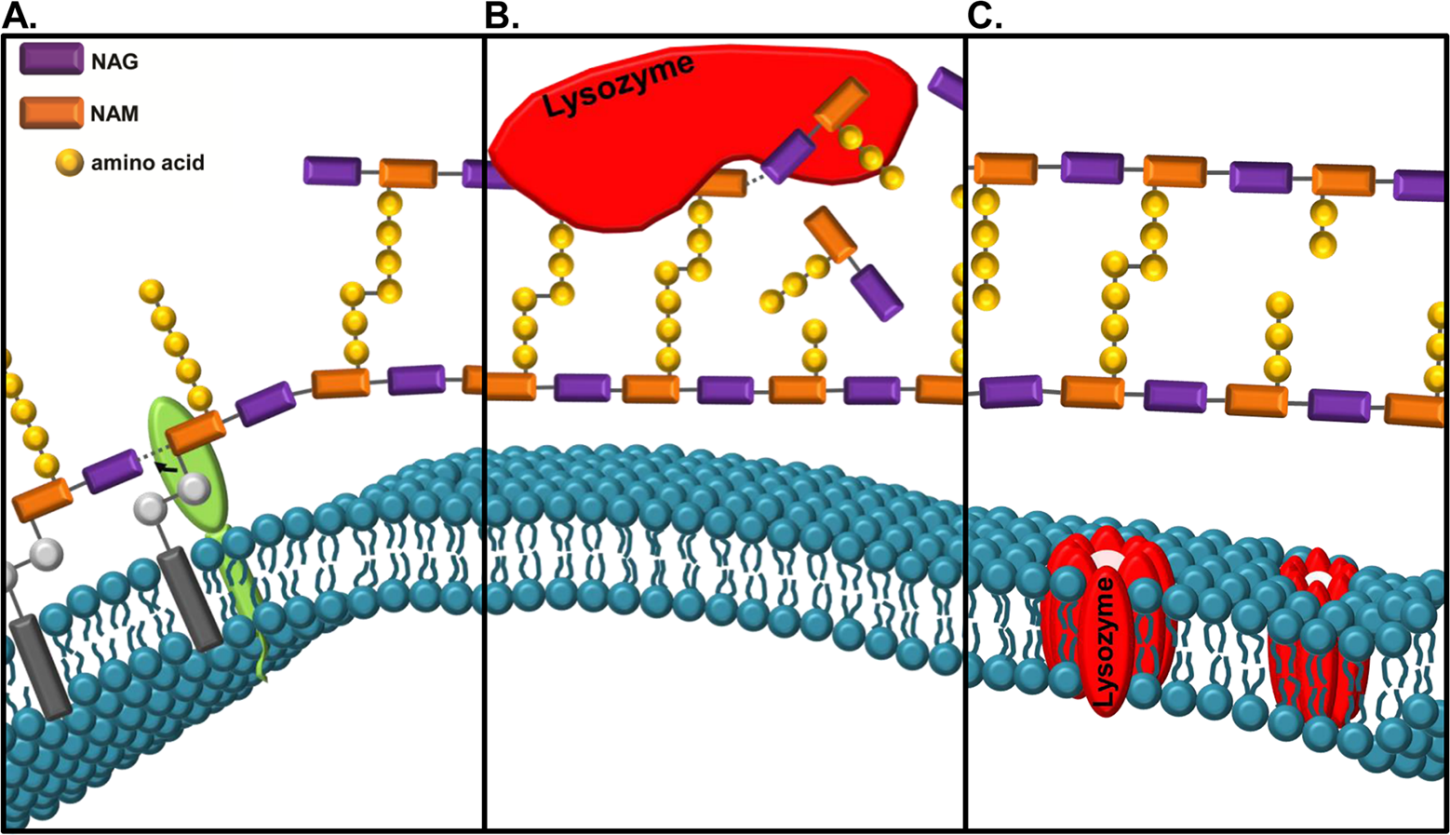
Lysozyme can kill bacteria through 2 mechanisms. (A) A newly synthesized PG monomer consists of a disaccharide, NAG linked to NAM with an attached peptide stem, and the NAM is anchored to the membrane via a lipid carrier (grey). Monomers are added to a growing chain through the action of glycosyltransferases (green). (B) Lysozyme hydrolyzes the β-1,4 glycosidic bond between the NAM of 1 monomer and the NAG of the adjacent monomer. Lysozyme hydrolysis of PG leads to cell wall instability and bacterial cell death. (C) Lysozyme can also kill bacteria independently of PG hydrolysis through a mechanism involving its cationic nature. Cationic killing of bacteria may involve the formation of pores by lysozyme (red cylinders) on the bacterial cell membrane. Abbreviations: NAG, *N*-acetylglucosamine; NAM, *N*-acetylmuramic acid.

**Fig 2:**
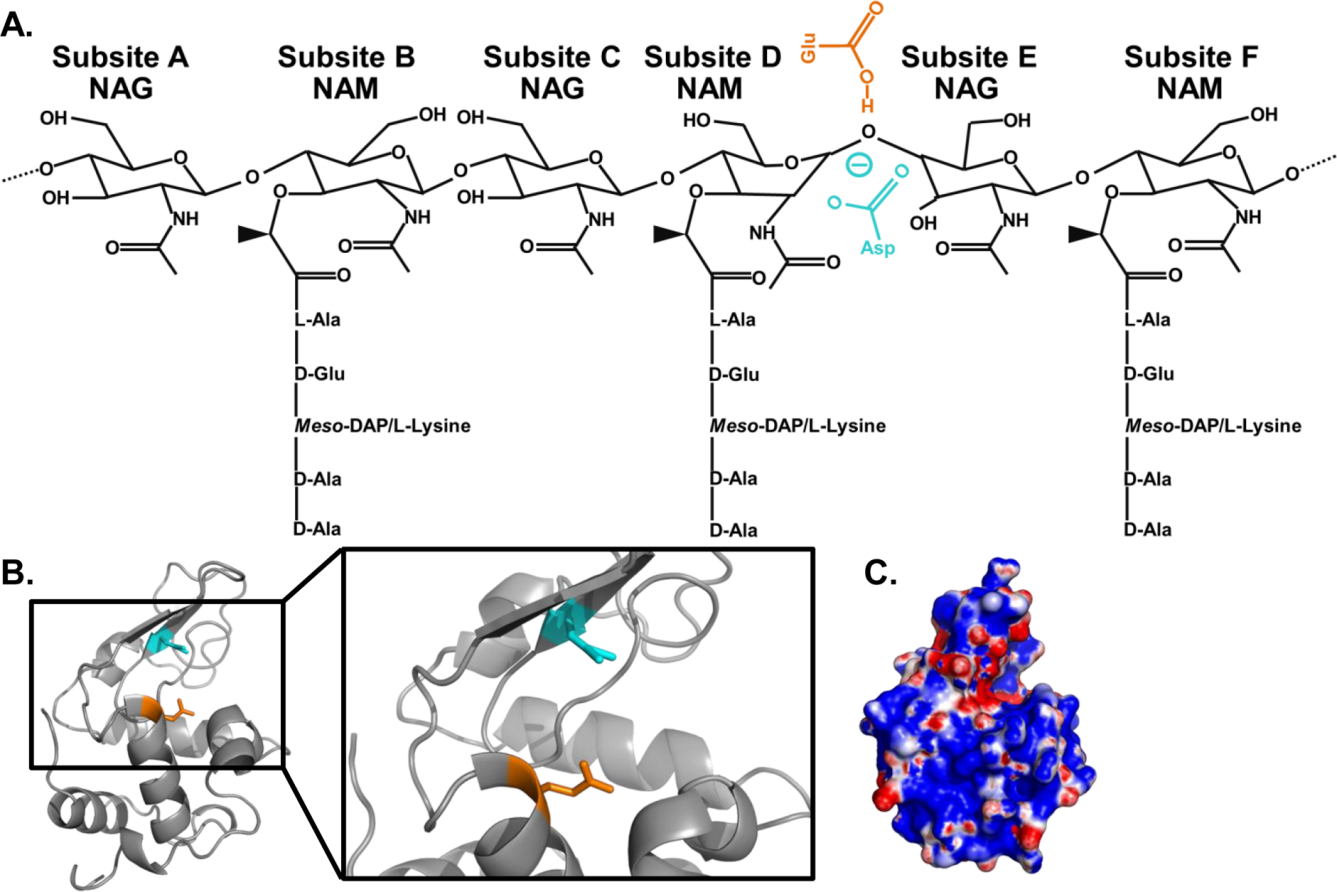
The enzymology and cationicity of human lysozyme. (A) The active site of lysozyme accommodates up to 6 consecutive sugars through 6 subsite contacts, annotated A-F. Lysozyme hydrolyzes the β-1,4 glycosidic bond between the NAM at subsite D and the NAG at subsite E [1]. (B) Ribbon model of human lysozyme highlighting the essential active site residues, an aspartic acid (blue) and a glutamic acid (orange). (C) Electrostatic potential map of human lysozyme (isoelectric point, 9.28). Because the bacterial envelope is negative, lysozyme may have an enhanced charge-mediated attraction for the bacterial surface that is proposed to lead to a catalytic-independent mechanism of bacterial killing. This structure was created using space-filling models in the PyMOL molecular graphics system. The electrostatic potential map was then calculated with the APBS Tools plug-in for PyMOL with default settings (contoured at ± 5kT/e; blue, positive; red, negative; white, hydrophobic). Human lysozyme, PDB accession 1REX. Abbreviations: NAG, *N*-acetylglucosamine; NAM, *N*-acetylmuramic acid; PDB, Protein Data Bank.

Nearly 100 years ago, Alexander Fleming was the first to observe the bacteriolytic efficacy of lysozyme [4]. We now know that lysozyme causes bacterial cell lysis via targeted hydrolysis of bacterial cell walls, which are critical for the resistance of bacteria to osmotic stress. The cell wall, or sacculus, is composed of PG monomers consisting of the disaccharide *N*-acetylglucosamine (NAG)-*N*-acetylmuramic acid (NAM) with a peptide stem attached to the lactyl moiety of NAM (Fig 1A). PG polymers are formed by β-1,4 glycosidic linkages between the NAM and NAG of individual monomers, and, to confer tensile strength to the sacculus, peptide stems are crosslinked between adjacent PG polymers. Variations in the structure and synthesis of PG amongst different bacteria have been reviewed elsewhere, but one notable difference is that gram-positive bacteria have a thick layer of PG that is exposed extracellularly, whereas the PG of gram-negative bacteria is thinner and sandwiched between the inner and outer membranes [5, 6]. Lysozyme hydrolyzes the β-1,4 glycosidic bond that links adjacent monomers (Figs 1B, 2A and 2B). Lysozyme’s enzymatic activity is covered in [1]. In addition to their enzymatic activity, c-type lysozymes, like human lysozyme and mouse LysM and LysP, are cationic (Fig 2C) [1, 7] and can insert into and form pores in negatively charged bacterial membranes (Fig 1C) [8, 9]. Both the enzymatic and cationic features of c-type lysozyme have been implicated in antibacterial activity [1, 7–10].

Given the abundance and potent activity of lysozyme against bacteria, it is not surprising that pathogenic bacteria have developed mechanisms to resist killing by lysozyme. In addition, the antimicrobial function of lysozyme is coupled with an important immunomodulatory role because components released from bacteria in a lysozyme–dependent manner can alter innate immune function. In this review, we will highlight the diverse and complementary mechanisms that pathogenic bacteria use to resist killing by c-type lysozyme, the effect of lysozyme on immune–mediated outcomes of infection, and the interplay between these features, with a focus on recent findings in each of these areas. We will then synthesize these findings in light of an overall model that places lysozyme as a key modulator of host-pathogen dynamics.

## Mechanisms of bacterial lysozyme resistance

Bacteria with the potential to cause disease have evolved the following 3 broad approaches to evade killing by lysozyme: modifying PG to render it resistant to hydrolysis by lysozyme, altering bacterial envelope charge and integrity, and expressing inhibitors of lysozyme.

### PG modifications

In order to bind its PG substrate, lysozyme must properly orient its active site residues with the glycan backbone of PG (Fig 2) [1]. Three types of PG modifications prevent the effective binding of lysozyme to PG: *N*-deacetylation of NAG and both *O*-acetylation and *N*-glycolylation of NAM. See Davis and Weiser for a historical perspective on these modifications [11]. Less common PG modifications that block hydrolysis by lysozyme are also described.

#### Deacetylation of NAG

**Fig 3:**
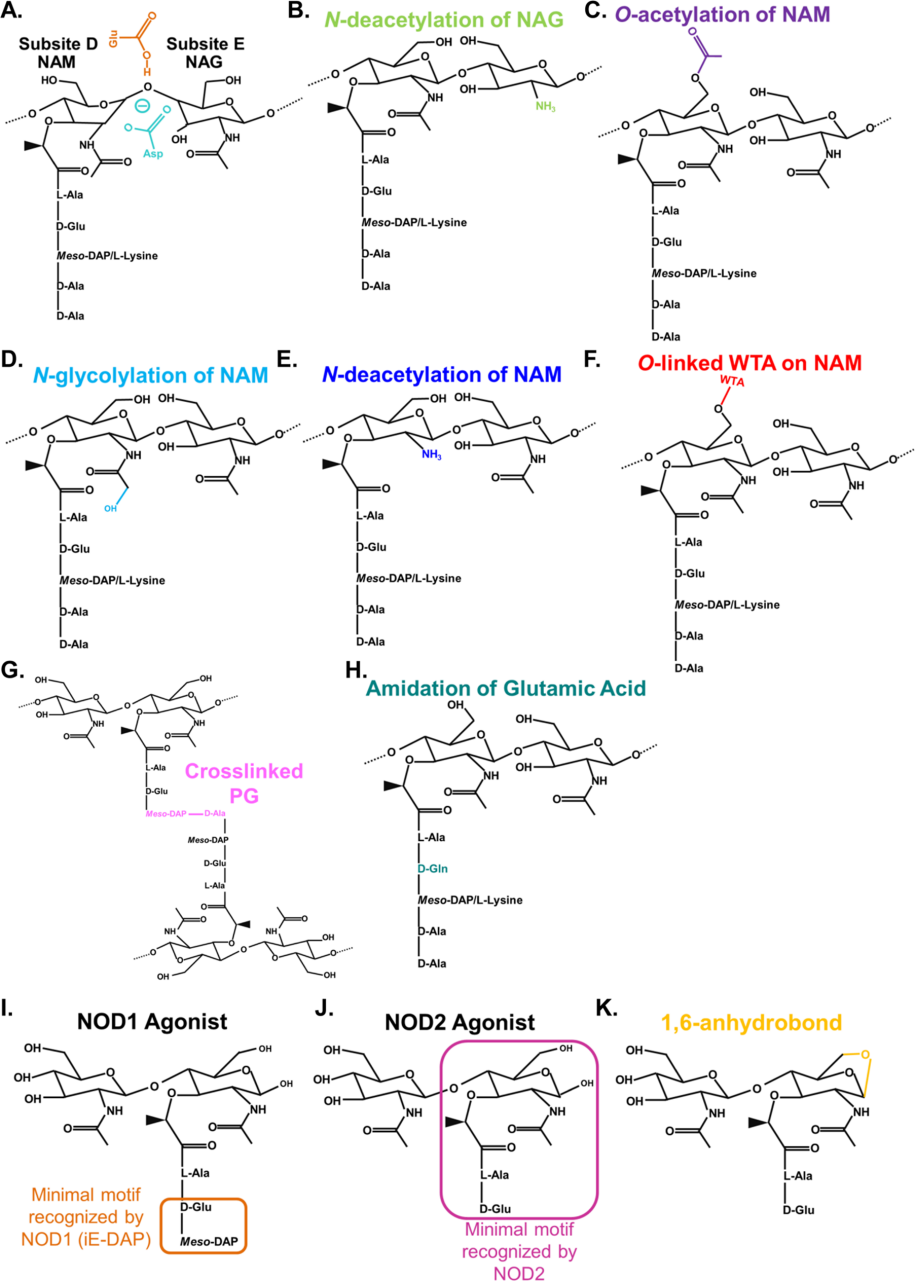
Bacteria modify PG to increase resistance to lysozyme, and some modifications can affect downstream innate detection. To disrupt efficient lysozyme binding to PG (A), bacteria modify their PG via *N*-deacetylation of NAG (B), *O*-acetylation of NAM (C), and *N*-glycolylation of NAM (D). Bacteria that *N*-deacetylate NAM (E), add WTAs to NAM (F), highly crosslink their PG (G), or amidate D-glutamic acid (H) are also more resistant to lysozyme. NAM on PG fragments that are released by lysozyme are in a reduced form and can activate the pattern recognition receptors NOD1 (I) and NOD2 (J). In contrast, PG released by bacterial lytic transglycosylases occurs with the formation of a 1,6-anhydrobond on the NAM residue, which can prevent NOD2 detection (K). *N*-deacetylation of NAM and *N*-glycolylation of NAM can decrease and increase NOD2-PG detection, respectively, whereas *O*-acetylation of NAM does not affect NOD2-PG detection. Abbreviations: NAG, *N*-acetylglucosamine; NAM, *N*-acetylmuramic acid; PG, peptidoglycan; WTAs, wall teichoic acids.

Interactions between the active site of lysozyme and the acetyl groups on the glycan backbone of PG facilitate efficient hydrolytic activity [1]. To limit these interactions, some pathogenic bacteria express a NAG deacetylase, encoded by *pgdA*, which removes the acetyl group at the C2 position of NAG (Fig 3B) [12]. *Streptococcus pneumoniae* lacking *pgdA* is more sensitive to killing by lysozyme in vitro and is less virulent in vivo [12, 13]. *pgdA* homologs in other bacteria, including *Helicobacter pylori*, *Listeria monocytogenes*, *Streptococcus suis*, *Streptococcus iniae* (*pdi*), *Enterococcus faecalis*, *Shigella flexneri*, *Mycobacterium tuberculosis* (*Rv1096*), and *Clostridium difficile* (*pdaV*), enhance bacterial resistance to lysozyme in vitro, increase bacterial survival in vivo, and/or increase bacterial survival in the presence of professional phagocytes [14–22]. Because the outer membrane of gram-negative bacteria occludes the passage of molecules that are larger than 650 Da [23], including lysozyme (e.g., human lysozyme is 14.7 kDa), *pgdA* mutants in the gram-negative *H*. *pylori* and *S*. *flexneri* are sensitive in vitro to lysozyme only upon the addition of a membrane-disrupting agent such as lactoferrin. Because *pgdA* mutants in these species also display decreased survival in vivo, it implies that there are membrane-disrupting conditions that increase sensitivity to lysozyme during infection [17, 24, 25].

#### Acetylation of NAM

The addition of an acetyl group to the C6 hydroxyl group of NAM, termed *O*-acetylation, prevents the binding of lysozyme to PG through steric hindrance (Fig 3C) [26]. *O*-acetylation of NAM is a common bacterial modification, although the mechanism occurs differently between gram-negative and gram-positive bacteria [27]. In the gram-positive bacterium *Staphylococcus aureus*, NAM acetylation by *O*-acetyltransferase A (*oatA*) enhances resistance to lysozyme in vitro and bacterial survival in vivo [28, 29]. The loss of the NAM *O-*acetyltransferase in *L*. *monocytogenes* (*oatA*), *S*. *pneumoniae* (*adr*), and *Bacillus anthracis* (*oatB*) renders these gram-positive bacteria sensitive to lysozyme, but only if they also lack *pgdA* or have fully *N*-acetylated PG [30–32]. Similarly, *E*. *faecalis* requires deletions in multiple lysozyme resistance factors, including *pgdA* (*EF1843*) and *oatA* (*EF0783*) before lysozyme sensitivity is observed [18, 19]. In contrast, *Lactobacillus plantarum O*-acetylates both NAM (via OatA) and NAG (via OatB), but only NAM *O*-acetylation confers resistance to lysozyme [33]. Thus *N-*deacetylation of NAG and *O*-acetylation of NAM can work additively but are not necessarily equivalent in their contribution to lysozyme resistance in gram-positive bacteria, implying that additional species-specific features contribute to lysozyme resistance. Furthermore, the redundancy, potency, and conservation of these resistance factors underscore the importance of lysozyme resistance to bacterial pathogenesis.

Conversely, in gram-negative bacteria, *O*-acetylation of NAM requires the following gene products: PatA (or PacA), a transmembrane protein that transports acetate from the cytoplasm to the periplasm, and PatB (or PacB), the periplasmic *O-*acetyltransferase [27]. In *H*. *pylori* and *Campylobacter jejuni*, the deletion of *patA* increases bacterial susceptibility to lysozyme in the presence of lactoferrin [24, 34]. In *C*. *jejuni*, *patA* mutant bacteria are more sensitive to killing by macrophages in vitro and have a decreased capacity to colonize the intestine in vivo [34]. In *Neisseria gonorrhoeae* and *Neisseria meningitidis*, *pacA* and *pacB* are important for PG *O*-acetylation and the resistance of purified PG to lysozyme [35]. We recently reported that *pacA* does not affect the sensitivity of *N*. *gonorrhoeae* to lysozyme unless bacterial envelope integrity is also compromised [36]. The loss of *pacA* did not increase gonococcal sensitivity to killing by human neutrophils, which implies that the many mechanisms used by *N*. *gonorrhoeae* to resist killing by neutrophils are sufficient to protect the PG cell wall from lysozyme-mediated degradation [36, 37]. Notably, while lysozyme is abundantly produced by neutrophils, the contribution of lysozyme to the killing of bacteria, including *N*. *gonorrhoeae*, in neutrophils remains unresolved.

It is noteworthy that *O-*acetylation of NAM is important in bacterial physiology beyond lysozyme inhibition, and this modification can also inhibit the activity of lytic transglycosylases, which are bacteria-derived cell wall turnover enzymes (see “Gram-negative envelope integrity” section) [38]. In *E*. *faecalis*, PG is basally modified with *O*-acetyl groups, whereas other PG modifications, including *N*-deacetylation of NAG, only occur when the bacteria are exposed to lysozyme [18, 19]. A lack of *O*-acetylation can increase autolysis and/or prevent cell separation, as is the case for *B*. *anthracis* [32]. On the other hand, excessive *O-*acetylation in *N*. *meningitidis* and *C*. *jejuni*, which is caused by the loss of the PG *O-*acetyl esterase Ape1, causes decreased bacterial virulence in vivo [39, 40]. While these observations indicate that *O*-acetylation is important for bacterial physiology as well as in resistance to lysozyme, the interplay between these functions in the context of infection remains unclear.

Moreover, the expectation that gram-positive bacteria use OatA while gram-negative bacteria use PatA/PatB for PG *O-*acetylation is not so simple. The gram-positive *B*. *anthracis* possesses homologs of both systems, with both contributing to PG *O*-acetylation. However, the systems do not appear to be redundant because only PatA/PatB but not OatB contributes to cell separation [32]. Future studies should reveal if other bacteria harbor multiple *O-*acetylation system, and, if so, how these systems respectively contribute to lysozyme resistance and/or bacterial physiology, possibly through differential spatial or temporal distribution in the bacterial cell.

#### *N-*glycolylation of NAM

Mycobacteria and some closely related Actinomycetes *N-*glycolylate NAM (Fig 3D). In mycobacteria, the production of *N*-glycolylmuramic acid is catalyzed by the hydroxylase NamH, a monooxygenase enzyme. The loss of *namH* in *Mycobacterium smegmatis* results in a decreased resistance to lysozyme [41]. Compared with NAG deacetylation or NAM *O-*acetylation, relatively few bacterial species analyzed to date *N-*glycolylate their PG. This may be related to the fact that PG with *N*-glycolylated NAM are better recognized by the host pattern recognition receptor NOD2, resulting in an enhanced pro-inflammatory response (see “Effects of lysozyme on innate detection of PG through NOD1 and NOD2” section) [42, 43].

#### Cell wall crosslinking and other modifications to PG structure

Additional modifications to the glycan backbone as well as crosslinking of the peptide stem of PG impede the ability of lysozyme to catalyze PG hydrolysis. *Bacillus subtilis* produces a polysaccharide deacetylase, PdaC, which *N*-deacetylates NAM in the context of intact PG and can also *N-*deacetylate NAG in short (NAG)_*n*_ oligomers (Fig 3E) [44]. A *pdaC* mutant has an increased sensitivity to lysozyme, but whether this phenotype is attributable to the enzymatic activities of PdaC on NAG or NAM or both has not yet been resolved [44]. In *S*. *aureus*, a wall teichoic acid can be covalently coupled to the C6 hydroxyl group of NAM, and like *O-*acetylation at this position, this contributes to an increased resistance to lysozyme, presumably via steric hindrance (Fig 3F) [45]. However, the addition of wall teichoic acids to PG could also increase lysozyme resistance by affecting the degree of PG crosslinking and thus the accessibility of lysozyme to its substrates [46]. In support of this latter possibility, mutants in *S*. *pneumoniae murMN* have fewer PG peptide crosslinks and are more sensitive to lysozyme as well as to nonenzymatic, cationic antimicrobial peptides [47]. In *S*. *aureus*, inhibiting PG crosslinking via penicillin has no effect on wild-type bacteria but increases lysozyme sensitivity in an *oatA* background (Fig 3H); whether PG crosslinking in *S*. *aureus* directly inhibits the enzymatic activity of lysozyme or causes pleiotropic effects that increase sensitivity to lysozyme has not been resolved [45]. Other evidence supporting a role for general cell wall remodeling in lysozyme resistance is demonstrated by work on both *L*. *monocytogenes* and *B*. *subtilis*, in which the putative penicillin-binding protein PbpX is required for lysozyme resistance, although through an as yet uncharacterized mechanism [48, 49].

### Alterations in envelope charge and envelope integrity

Cationic antimicrobial proteins are highly attracted to the negatively charged cell envelope of bacteria, and this interaction is important for efficient bacterial killing [10]. Therefore, changes to the bacterial envelope that reduce the net negative charge toward a more neutral charge can concomitantly reduce the binding of lysozyme as well as other cationic antimicrobial proteins of host defense.

#### PG charge

The PG sacculus itself has a net negative charge [50]. In *S*. *aureus*, MurT and GatD amidate glutamic acid to glutamine at the second position in the PG peptide stem, consequently reducing the net negative charge of PG (Fig 3G) [50–52]. The amidation of glutamic acid increases the resistance of intact bacteria and purified PG to lysozyme [51, 52]. *Lactococcus lactis* amidates its aspartic acid crossbridge residues via a putative asparagine synthase (*asnH*), which increases *L*. *lactis* resistance to lysozyme [53]. In addition, *N*-deacetylation of NAG, which perturbs enzymatic hydrolysis by lysozyme, also results in PG with a reduced negative charge. It is still unclear whether modifications that affect the charge of PG strictly alter cationic killing by lysozyme or whether such modifications exert pleiotropic effects that affect lysozyme resistance. Furthermore, because positively charged residues in the active site of lysozyme are important for substrate recognition, it is possible that perturbing the PG charge could alter substrate recognition by lysozyme and hence thereby affect its enzymatic activity [54].

#### Teichoic acid charge

In *S*. *aureus*, D-alanylation of teichoic acids by the *dlt* operon also reduces the net negative charge of the cell envelope [55]. *S*. *aureus* lacking *dltA* is more sensitive to killing by cationic antimicrobial proteins, including lysozyme, in vitro, and lysozyme-mediated killing of *dltA* mutants occurs independently of its enzymatic activity [55, 56]. Similar results have been found for other bacteria that add D-alanine to teichoic acid [48, 49, 57–60]. However, not all changes to cell envelope charge are equivalent, because the mutation of *dltA* in *S*. *suis* results in an increased sensitivity to some cationic antimicrobial proteins but not to lysozyme [61]. This may imply that other modes of defense against lysozyme are more important to *S*. *suis* pathogenesis.

#### Lipid charge

Reducing the negative charge of plasma membrane lipids can be an effective defense against cationic antimicrobial proteins, including lysozyme. For instance, the MprF family of enzymes lysinylate polar membrane lipids. The MprF homolog in *M*. *tuberculosis*, LysX, contributes to resistance to lysozyme as well as to cationic dyes and antimicrobial proteins and enhances bacterial survival in macrophages [62, 63]. However, if bacteria have redundant, compensatory mechanisms that can maintain an advantageous envelope charge, the loss of *mprF* may have a limited effect on susceptibility to lysozyme, as observed in *E*. *faecalis* [57].

The negatively charged molecule lipopolysaccharide in the outer membrane of gram-negative bacteria can attract cationic antimicrobial proteins [64]. In fact, the presence of lipopolysaccharide in in vitro–generated lipid monolayers is sufficient to promote the insertion of lysozyme [65]. However, while reducing the negative charge of lipopolysaccharide generally increases the resistance of gram-negative bacteria to cationic antimicrobial proteins [64], the degree to which this affects gram-negative resistance to lysozyme is largely unknown. In one example, in *Acinetobacter baumannii*, mutations that enhance the activation of the 2-component signal transduction system PmrAB increase the addition of phosphoethanolamine to the lipid A portion of lipopolysaccharide, resulting in a reduced negative charge [66]. Bacteria with these mutations have an increased resistance to lysozyme and to other cationic antimicrobial peptides [67]. We are currently examining in *Neisseria gonorrhoeae* how phosphoethanolamine addition to the lipid A of lipooligosaccharide by the enzyme LptA affects resistance to lysozyme because this modification enhances the resistance to killing by other cationic antimicrobial proteins and by neutrophils [68, 69].

#### Gram-negative envelope integrity

In general, the gram-negative outer membrane prevents larger molecules like lysozyme from gaining access to interior targets [23]. The extent of intrinsic barrier function of the outer membrane varies among species and can be perturbed by changes to the integrity of the cell envelope [23]. For instance, we recently reported that 2 PG-recycling enzymes, the lytic transglycosylases LtgA and LtgD, are important for lysozyme resistance in *N*. *gonorrhoeae* by contributing to envelope integrity [36]. The related cell wall–recycling homologs in *Escherichia coli* similarly contribute to envelope integrity and lysozyme resistance [70]. In *N*. *gonorrhoeae*, cell envelope integrity and resistance to lysozyme by LtgA and LtgD is correlated with increased likelihood of survival in the presence of human neutrophils [36]. This observation suggests that inhibiting lytic transglycosylase activity, for instance through the antibiotic bulgecin A [71], could effectively combat infections with gram-negative bacteria by reducing envelope integrity and consequently enhancing their sensitivity to killing by lysozyme and potentially other innate immune components.

Cationic antimicrobial peptides such as lactoferrin synergize with lysozyme for the enhanced killing of gram-negative bacteria through a proposed mechanism by which lactoferrin permeabilizes the outer membrane to enhance the access of lysozyme to periplasmic PG [25]. Lysozyme itself can form pores on bacterial membranes in some contexts [8, 9], yet it is still unclear if these pores are sufficient to enhance the transit of other lysozyme molecules to the periplasm to enzymatically degrade PG.

### Bacterial inhibitors of lysozyme

Some gram-negative bacteria, such as *Pseudomonas aeruginosa* and *E*. *coli*, are intrinsically resistant to lysozyme, yet these bacteria notably lack PG modifications like the *O*-acetylation of NAM [72, 73]. Instead, they express a periplasmic protein inhibitor of lysozyme that is termed Ivy [73–75]. Inhibition occurs through a loop protrusion in Ivy that occludes the active site of lysozyme via a lock-and-key mechanism [75]. Ivy is important for in vitro resistance to lysozyme for *E*. *coli* and *Yersinia pestis*. Furthermore, Ivy is important for the survival of *Y*. *pestis* from human neutrophils ex vivo and for optimal virulence in a mouse model of bubonic and pneumonic plague [76]. Other bacteria produce additional periplasmic lysozyme inhibitors such as MliC and PliC (reviewed in [77]). Although most examples place Ivy, MliC, and PliC in the periplasm, Humbert et al. recently found the surface-exposed adhesin complex protein of *Neisseria* spp. shares overall structural homology with MliC, and, like MliC, directly inhibits lysozyme activity [78].

In addition to inhibiting lysozyme, Ivy-type proteins also inhibit bacterial lytic transglycosylases [73]. As described above, lytic transglycosylase–mediated remodeling of the cell wall optimizes envelope integrity and contributes to the defense against lysozyme. However, unrestrained lytic transglycosylase activity can reduce PG tensile strength and lead to autolysis. Moreover, the PG fragments released by lytic transglycosylases may activate host pattern recognition receptors (see “Lysozyme activity modulates innate immune responses” section). Thus, Ivy-type inhibitors play complex and overlapping roles in gram-negative bacterial physiology, immune modulation, and host defense, and most studies have not discriminated among these functions. For instance, *Legionella pneumophila* EnhC inhibits the lytic transglycosylase SltL and enhances bacterial survival in association with macrophages [79]. While it was suggested that the mechanism for increased macrophage survival is to prevent the release of immunostimulatory PG fragments, EnhC may also defend against macrophage-derived lysozyme and/or assist in the optimal structuring of the *L*. *pneumophila* envelope [79]. Although bacterially encoded inhibitors of lytic transglycosylases have not been described for gram-positive bacteria, *B*. *subtilis* produces an inhibitor of PG endopeptidases, IseA; its contribution to lysozyme resistance has not been studied [80].

### Regulation of lysozyme resistance factors

While lysozyme resistance factors appropriately tailor the bacterial response to immune pressure, modifications to PG that increase resistance to lysozyme may have a fitness cost, as reported for *S*. *pneumoniae* [30]. Thus, it is not surprising that the expression of many of the factors described above is increased upon the exposure to lysozyme or to immune cells [14, 16, 18, 81]. For example, *E*. *faecalis* deacetylates NAG only after lysozyme challenge [18, 19], and MliC is up-regulated in *Salmonella enterica* serovar Typhi within macrophages [81]. Regulation of lysozyme resistance factors occurs both transcriptionally and posttranscriptionally.

#### Transcriptional regulation

In several gram-positive pathogens, the extracytoplasmic function sigma factor σ^V^ (gene, *sigV* or *csfV*) regulates genes with products that enhance PG resistance to lysozyme. σ^V^ is sequestered by the membrane-bound anti-sigma factor RsiV. The binding of lysozyme to RsiV leads to the degradation of RsiV and release of σ^V^ [49, 82–84]. In *B*. *subtilis*, σ^V^ regulates *oatA* and *dltA*, whereas in *C*. *difficile*, σ^V^ regulates *pdaV*, a PG *N*-deacetylase, and *dltA* [21, 49, 59, 82]. Despite possessing *oatA* and *dltA*, *E*. *faecalis* σ^V^ only appreciably regulates the expression of *pgdA* in response to lysozyme [57]. While σ^V^ in *B*. *subtilis* and *C*. *difficile* is specifically induced by lysozyme, σ^V^ from *E*. *faecalis* is induced by lysozyme as well as other cell wall stressors [21, 49, 82, 85]. *sigV* mutants in *B*. *subtilis*, *C*. *difficile*, and *E*. *faecalis* all have an increased sensitivity to lysozyme in vitro [21, 49, 59, 82, 85]. *E*. *faecalis sigV* mutants are decreased in bacterial burden in vivo, and *C*. *difficile sigV* mutants can have significantly attenuated virulence in vivo, depending on the experimental model [21, 57, 59].

The 2-component signal transduction system GraRS induces the expression of the *dlt* operon in *S*. *aureus* and enhances bacterial resistance to lysozyme [56]. GraRS is activated by specific cationic antimicrobial peptides, but the mechanism underlying this activation still remains to be elucidated [86]. Similarly, the 2-component system VirRS in *L*. *monocytogenes* positively regulates the *dlt* operon, and *virR* mutants are attenuated in vivo [60, 87]. In *S*. *iniae*, the transcriptional regulator CpsY is important for *O*-acetylation of NAM and an increased resistance to killing by lysozyme and human neutrophils by indirectly affecting *oatA* transcription [88, 89]. Similarly, SpxB in *L*. *lactis* directly binds to the α subunit of RNA polymerase to enhance the transcription of *oatA*, and SpxB activity is linked to the 2-component signal transduction system CesSR that responds to envelope stress [90]. In *S*. *enterica* serovar Typhimurium, the transcriptional regulator SlyA up-regulates the expression of a putative lysozyme inhibitor, PliC (STM1249), although the cues that induce SlyA activity in this context are unknown [91].

#### Post-transcriptional regulation

In *L*. *monocytogenes*, the small noncoding RNA Rli31 positively regulates *pgdA* and a putative PG peptidase, *pbpX*, while the RNA-binding protein SpoVG negatively regulates lysozyme resistance through an undefined mechanism [48, 92]. In *H*. *pylori*, oxidative stress and exposure to macrophages can induce the expression of the NAG *N-*deacetylase *pgdA* via posttranscriptional regulation by the apo-aconitase AcnB. *H*. *pylori* mutants lacking *acnB* have an increased sensitivity to lysozyme in vitro and a decreased ability to colonize the mouse stomach in vivo [14, 93, 94].

Given the number and diversity of lysozyme resistance factors in both gram-negative and gram-positive bacteria, much remains to be learned about how these factors are regulated. Because many nonpathogenic bacteria encode homologs of Ivy, PgdA, and Oat and Pat *O-*acetyltransferases [32, 44, 49, 53, 74, 82, 90], we speculate that pathogenic bacteria produce higher levels of lysozyme resistance factors or exert tighter control over the regulation of these factors than commensals, although these comparisons remain to be made. Future studies should aim to identify the factors involved with regulating lysozyme resistance genes and to characterize their mechanisms of regulation.

### Lysozyme activity modulates innate immune responses

Many studies testing the contribution of lysozyme to immune cell responses have relied upon mice that lack lysozyme M (gene, *lysM*). Lysozyme M is homologous to the single human lysozyme and is produced by phagocytes and other myeloid cells [95]. Mice also produce a second lysozyme, lysozyme P, which is expressed by intestinal Paneth cells. It has been shown that LysM^-/-^ mice can exhibit compensatory expression of lysozyme P in nonintestinal cells [7, 95]. Thus, the potential for compensatory expression of lysozyme P in LysM^-/-^ mice should be kept in mind in the context of the studies reviewed below; a mouse lacking both lysozyme M and P does not exist, to our knowledge.

### Lysozyme activates pro-inflammatory immune responses

Lysozyme produced by neutrophils and macrophages can be delivered to bacterium-containing phagosomes [1]. Accordingly, bacteria that are more sensitive to lysozyme are more likely to be degraded in the phagosomes of macrophages in a LysM-dependent manner [31, 96]. In human neutrophils, we recently demonstrated a correlation between the susceptibility of *N*. *gonorrhoeae* to lysozyme and enhanced neutrophil activation, as measured by increased granule release at the plasma membrane and into phagosomes, which illustrates that lysozyme may modulate immune activation in other phagocytes [36]. Pattern recognition receptors activated downstream of lysozyme-mediated degradation include the NOD1 and NOD2 receptors, Toll-like receptors (TLRs), and inflammasomes. The following sections will cover these inflammatory responses mainly in the context of phagocytes.

#### Effects of lysozyme on innate detection of PG through NOD1 and NOD2

PG is made by almost all bacteria but not by eukaryotes, making it an excellent target for pattern recognition receptors. The sensing of PG by the cytosolic receptors NOD1 and NOD2 stimulates downstream pro-inflammatory signaling events via the activation of NF- κB, including the production of pro-inflammatory cytokines such as interleukin (IL)8 and antimicrobial molecules [97, 98]. Notably, sufficient quantities of stimulatory PG can be released by lysozyme even when lysozyme does not markedly affect bacterial viability [96].

In humans, NOD1 recognizes PG-derived peptides containing D-glutamyl-*meso*-diaminopimelic acid (iE-DAP), making NOD1 a selective receptor for the detection of gram-negative bacteria, which predominantly incorporate this amino acid into PG (Fig 3I) [98]. Bacteria naturally release the tripeptide L-alanine-D-glutamyl-*meso*-diaminopimelic acid, not iE-DAP, during normal cell wall turnover, and the tripeptide, attached or not to NAM, stimulates NOD1 to a greater extent than iE-DAP alone [99–101]. To date, no modifications that affect the ability of lysozyme to hydrolyze the glycan backbone of PG are implicated in signaling via NOD1; however, NOD1 recognition is reduced by alterations in the PG peptide stem that also affect susceptibility to lysozyme, such as *N-*myristoylation or the amidation of glutamic acid [97, 101].

NOD2 recognizes NAM with an attached dipeptide stem (i.e., muramyl dipeptide, or MDP), which is produced by both gram-negative and gram-positive bacteria (Fig 3J) [98]. The recognition of MDP by NOD2 is direct, and the presence of NAM in MDP is vital for effective NOD2-MDP recognition [101–104]. The biochemical properties of MDP and NOD2 that contribute to ligand binding and downstream NOD2 signaling are reviewed in [98, 105]. NOD2 has yet to be crystalized in complex with MDP, but it is predicted that NOD2 interacts with the peptide stem and proximal carbons of NAM (e.g., C2) [106]. Modifications to PG that alter lysozyme-mediated hydrolysis also affect NOD2-MDP recognition and NOD2 signaling. In particular, *N*-glycolylation of NAM on the proximal C2 *N*-acetyl group enhances MDP recognition by NOD2 [42, 43, 107], while *N*-deacetylated NAM at the C2 position, as in PdaC-expressing *B*. *subtilis*, abrogates it [44, 107]. In contrast, the addition of a stearoyl fatty acid to the C6 distal *O*-acetyl group in NAM does not inhibit NOD2 signaling and in fact enhances it by allowing for the direct cytosolic entry of MDP [108].

The release of PG monomers by lysozyme is an important prerequisite to NOD2 activation. Chemically synthesized PG moieties of differing glycan lengths (e.g., tetrasaccharide, octasaccharide, etc.) have been used to show that smaller PG moieties are more stimulatory to NOD2 [102, 109]. The cell wall of *L*. *monocytogenes*, which intrinsically has *N*-deacetylated NAG, is a poor activator of NOD2 in HEK293 epithelial cells unless it is predigested with mutanolysin, which hydrolyzes PG in the same way that lysozyme does but is unaffected by this modification [15]. The cell wall from the *L*. *monocytogenes pgdA* mutant, which has acetylated NAG, was more stimulatory to NOD2 than the wild-type (WT) cell wall but markedly less so than predigested PG [15]. Notably, while lysozyme-derived PG monomers can stimulate NOD2, PG monomers derived from bacterial lytic transglycosylases poorly stimulate NOD2 (Fig 3K) [110, 111].

NOD1 is broadly expressed in a variety of cell types, including epithelial cells, and thus contributes to pro-inflammatory signaling in these cell types [98]. The expression of NOD1 is relatively low in phagocytes, but NOD1 has been implicated in altering phagocyte function in vivo, although it is still unclear whether this is driven by a phagocyte-specific NOD1 response [112–114]. In contrast, NOD2 expression is largely restricted to phagocytes and some specialized cell types, such as intestinal Paneth cells [98, 114–116]. In phagocytes, the current working model for the activation of NOD family receptors posits that bacteria are phagocytosed and directed into lysosomes containing lysozyme and other antimicrobial components. There, intact, insoluble PG is processed into PG fragments in a lysozyme-dependent manner. PG monomers are then transported across the endosomal membrane via SLC15 family peptide transporters to NOD proteins, which dock on the cytosolic face of the endosome [96, 117, 118]. Phagocytes appear to be optimized to respond to phagosomally produced PG fragments, not extracellular ones, because peripheral monocytes and neutrophils are poorly responsive to extracellular MDP [119], and macrophages only macropinocytose soluble MDP at high concentrations of ligand [120]. Further testing of this model has proven challenging because primary phagocytes are poorly genetically manipulable and bear limited resemblance to the favored model for NOD biology, the immortalized HEK293 cell line in which NOD proteins are overexpressed. Unlike phagocytes, HEK293 cells can detect exogenous, soluble PG, which bypasses the need for phagosomal processing [116, 119].

Taken together, these data show that the ability of lysozyme to digest PG alters the production of ligands that are recognized by NODs. Two of the remaining outstanding questions in this field that are germane to this review include defining what structures of PG are ultimately recognized by NODs in phagocytes and how modifications that alter lysozyme-mediated processing manipulate that recognition.

#### Lysozyme and activation of TLRs and inflammasomes

The lysozyme-mediated degradation of bacteria enhances the release of immunomodulatory bacterial products, including but not limited to PG. For example, lysozyme-sensitive *S*. *aureus* is more susceptible to degradation by macrophages, which is correlated with increased inflammatory cytokine production, such as TNFα and IL6, via TLR2 and TLR9, the receptors for bacterial-derived lipoproteins and DNA, respectively [121]. Similarly, lysozyme-sensitive *L*. *monocytogenes* induces the release of inflammatory cytokines, including type I interferons, from macrophages, by a pathway that is dependent on TLR2 [15].

**Fig 4:**
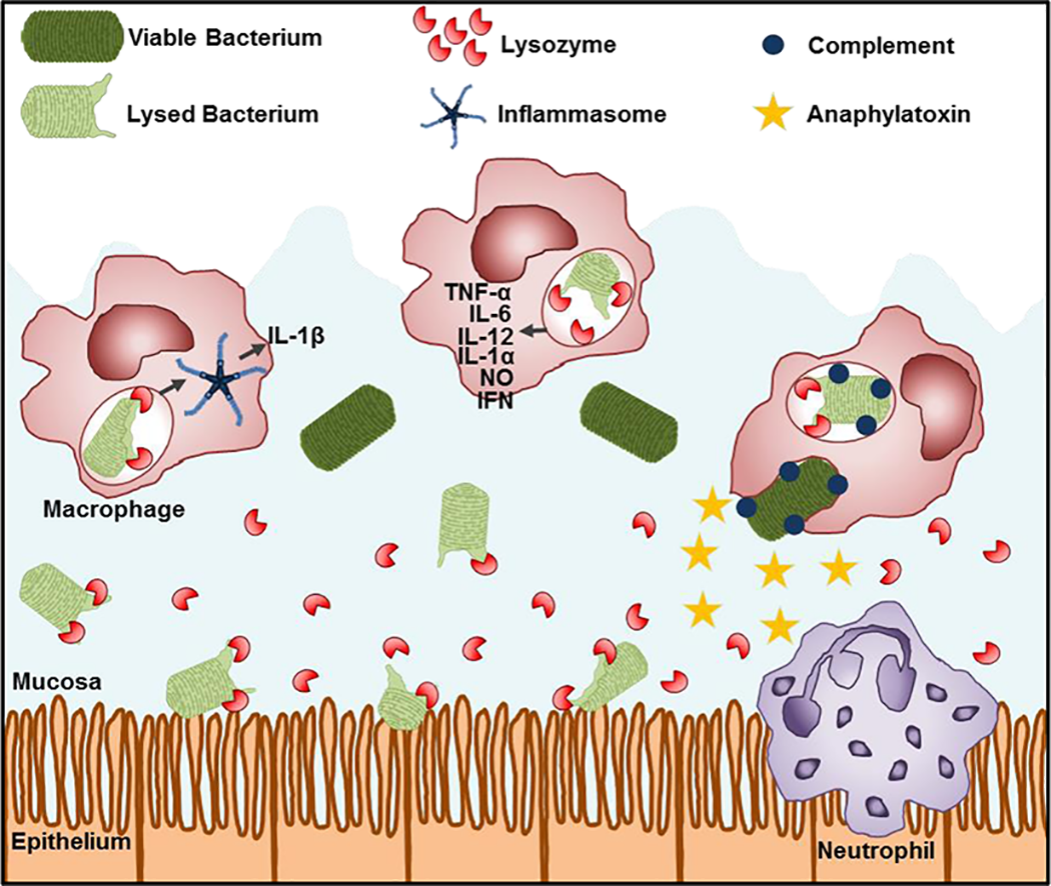
Lysozyme modulates the immune response. At the site of infection, extracellular lysozyme (red sector), which is secreted locally by the epithelium, can kill bacteria, leading to the release of PAMPs, including but not limited to monomeric PG. This can initiate an epithelial-driven response that leads to phagocyte recruitment (not depicted here). Resident or recruited macrophages also secrete lysozyme extracellularly and can internalize bacteria, delivering lysozyme to the bacterium-containing phagosome. In macrophages, bacterial degradation by phagosomal lysozyme releases PAMPs that stimulate a robust proinflammatory cytokine response and activate the inflammasome. Neutrophil activities may be similarly enhanced by lysozyme-mediated degradation of phagosomal bacteria, akin to macrophages. Deposition of complement (blue circles) on particles, including bacteria and/or insoluble polymeric PG, enhances bacterial phagocytosis and also produces complement-derived anaphylatoxins (yellow stars) that are chemotactic for phagocytes. Because phagocytes poorly respond to extracellular, monomeric PG and monomeric PG cannot activate complement, the degradation of bacterial PG by extracellular lysozyme serves to restrict phagocyte activation and recruitment. Thus, lysozyme activity can function to enhance or dampen the immune response. Abbreviations: PAMP, pathogen-associated molecular pattern; PG, peptidoglycan.

Frequently, the increased pro-inflammatory response of macrophages occurs via an increased activation of the inflammasome, resulting in IL1β secretion (Fig 4) [29, 31, 121–124]. PG and/or NOD2 activity has been implicated in stimulating the NLRP3 inflammasome as well as the NLRP1 and AIM2 inflammasomes [31, 125–128]. When lysozyme in macrophages is inhibited by using exogenously added NAG polymer (i.e., triNAG) or in a LysM^-/-^ background, PG induces less activation of the inflammasome [29, 31]. Reminiscent of the effects on NOD activation, in macrophages, insoluble PG but not soluble PG activates the inflammasome [29]. One direct mechanism for inflammasome activation by PG was recently elucidated by Wolf et al., who reported that NAG stimulates a pathway leading to re-localization of hexokinase from mitochondria to the cytosol, thereby activating NLRP3 [128]. *N*-deacetylation of NAG abrogates this response, linking PG modifications and, by extension, susceptibility to lysozyme-mediated degradation to NLRP3 activation [128]. Inflammasome activation may also be indirect through the lysozyme-catalyzed release of other stimulatory bacterial factors.

The increased lysozyme-mediated degradation of bacteria in phagocytes can lead to an overzealous inflammatory response. For example, macrophages that phagocytose *S*. *aureus* lacking *O*-acetylation have increased inflammasome activation in vitro, which correlates with increased lesion size in a subcutaneous skin infection model in vivo [29]. In this context, the inhibition of lysozyme with TriNAG reduced inflammasome activation in vitro [29]. Similarly, Müller et al. recently found that exposure to *S*. *aureus* harboring reduced numbers of PG crosslinks led to increased inflammasome activation in macrophages in vitro and increased lesion size in vivo, potentially owing to increased lysozyme digestion of under-crosslinked PG [124].

Together, these findings reveal that the lysozyme-mediated digestion of PG leads to the activation of multiple innate immune receptor families that stimulate pro-inflammatory responses. The location of lysozyme activity (particularly intracellular lysozyme), the susceptibility of PG to lysozyme digestion, and the amount and composition of the factors released as a consequence all modulate the degree and extent of innate immune activation.

### Contribution of lysozyme to the resolution of inflammation

Although lysozyme is important for driving a pro-inflammatory response, lysozyme also plays a role in limiting inflammation systemically, resulting in decreased inflammatory-driven pathology [7, 129]. LysM^-/-^ mice infected with *Klebsiella pneumoniae* by intratracheal injection have an increased bacterial burden but also produce less IL10, an anti-inflammatory cytokine, compared with WT mice [130]. Similarly, in an otitis media infection model with *S*. *pneumoniae*, LysM^-/-^ mice experienced enhanced inflammation compared with WT mice, which was concomitant with decreased bacterial clearance [131]. However, these studies did not distinguish between lysozyme functioning to directly limit inflammation and lysozyme-limiting bacterial outgrowth, which itself is pro-inflammatory. Addressing this issue, Ganz et al. subcutaneously injected mice with heat-killed *M*. *luteus* or purified PG from *M*. *luteus* and found that lesion size and immune infiltrates were increased in the absence of LysM [129]. These results demonstrate that the failure of lysozyme to clear PG is sufficient to drive increased inflammation, but lysozyme may still reduce inflammation by restricting bacterial growth.

Lysozyme also functions to limit intestinal inflammation. Using a murine model of Crohn’s disease, Zhang et al. and Wang et al. recently showed that intestinal inflammation is correlated with the failure of Paneth cells to sort and secrete lysozyme P, which is dependent on NOD2 and RIP2 [115, 132]. Furthermore, the addition of lysozyme to mice with dextran sodium sulfate–induced colitis can ameliorate intestinal inflammation [133]. There are several mechanisms that could explain how lysozyme limits inflammation. Lysozyme could assist in intestinal epithelial barrier protection to limit the invasion of the microbiota, which are normally not pathogenic unless they breach the epithelial barrier; liberate PG fragments that activate a protective intestinal immune response; or clear polymeric PG that could hyper-activate resident macrophages. Studies designed to test among these possibilities have not yet been performed.

The addition of exogenous lysozyme has a variety of other immune-dampening effects. It decreases chemotaxis and the production of an oxidative burst in neutrophils by as-yet unknown mechanisms [134, 135]. Lysozyme can directly bind and neutralize extracellular, prooxidant bioreactive derivatives, which are termed advanced glycation end products and are otherwise pro-inflammatory [136, 137]; however, this interaction also blocks the enzymatic bactericidal activity of lysozyme, which could have secondary effects on immune responses during infection [137]. Finally, extracellular, insoluble PG can trigger potent phagocyte chemotaxis via complement factors C3a and C5a, which are produced when complement is fixed onto insoluble PG. The lysozyme-mediated digestion of PG into soluble fragments reduces the production of these anaphylatoxins, thereby reducing phagocyte influx and concomitant cellular inflammatory responses (Fig 4) [138]. Taken together, these findings indicate that lysozyme contributes in multiple ways to resolve phagocyte-driven inflammation.

## Working model for the contribution of lysozyme to immune responses

The degradation of bacteria by lysozyme serves 2 purposes: (1) to kill bacteria and (2) to release immunomodulatory bacterial ligands, including PG fragments. Several recent studies have uncovered the mechanisms used by host organisms to detect bacterial PG as well as the numerous and complementary ways that bacteria evade recognition and degradation by lysozyme. Pathogenic bacteria that modify their cell surface, including alterations to the composition and crosslinking of PG can avoid degradation by lysozyme, thereby modulating both bacterial virulence and corresponding host responses. Knowledge of these mechanisms can illuminate avenues for novel antibacterial therapies, such as interfering with the ability to synthesize PG modifications that contribute to lysozyme resistance.

One intriguing conundrum that still remains to be resolved is the dual, potentially contradictory role of lysozyme in the immune response to infection. While the lysozyme-mediated degradation of PG enhances phagocyte activation to drive bacterial killing and the production of inflammatory mediators, lysozyme also helps to resolve inflammation. Based on these findings, we posit a model where lysozyme activities must be balanced temporally and spatially to appropriately tune immune responses during the course of infection (Fig 4).

Lysozyme plays an important role in limiting bacterial growth at mucosal surfaces and other sites, where it may not only control potentially pathogenic bacteria but also limit overgrowth of the microbiota to prevent dysbiosis. Extracellular lysozyme also degrades multimeric PG into soluble fragments that activate NOD receptors in mucosal epithelial cells, leading to the secretion of chemotactic and activating factors for neutrophils and macrophages. These phagocytes engulf bacteria into phagosomes that contain lysozyme and other degradative enzymes, which liberates PG fragments and other microbial-associated molecular patterns that further activate pro-inflammatory pathways.

Concomitantly, extracellular, soluble PG fragments are not particularly effective at activating phagocytes, and thus extracellular lysozyme limits the extent of phagocyte activation via PG. Moreover, extracellular lysozyme limits complement deposition on PG (either the gram-positive bacterial surface or insoluble PG fragments released from bacteria that are susceptible to extracellular digestion), thus reducing anaphylatoxin production and the recruitment of more phagocytes. If these activities of lysozyme occur later in the course of infection, they are likely to be important for the resolution of infection. A corollary of this model is that when bacteria are relatively resistant to lysozyme or if lysozyme abundance or activity is reduced, both lysozyme-mediated antibacterial defense and immune resolution are altered. Depending on the circumstance, this not only enhances the survival of pathogenic bacteria but also promotes a sustained inflammatory response with the potential to cause collateral tissue damage. Results from ongoing and future studies that seek to understand when, where, and how lysozyme is released and how pathogenic bacteria regulate the expression of lysozyme resistance mechanisms will refine this model to contextualize lysozyme as a critical and abundant agent of host immune defense.
